# Keys to the species of Mydaeinae (Diptera: Muscidae) from China, with the description of four new species

**DOI:** 10.1093/jis/14.1.22

**Published:** 2014-01-01

**Authors:** Wan-Qi Xue, Xu Tian

**Affiliations:** 1 Institute of Entomology, Shenyang Normal University, Huanghe North Street 253, Huanggu District, Shenyang 110034, China; 2 Institute of Applied Ecology, Chinese Academy of Sciences, Shenyang 110016, China; 3 University of Chinese Academy of Sciences, Beijing 100049, China

**Keywords:** Chinese species, classification, Mydaea, Myospila

## Abstract

Four new species of Mydaeinae, *Mydaea franzosternita***n. sp.** , *Myospila apicaliciliola***n. sp.** , *Myospila maoershanensis***n. sp.** , and *Myospila subflavipennis***n. sp.** , are described and illustrated here for the first time. A key to the genus of Mydaeinae from China and keys to species of genera from Mydaeinae are provided.

## Introduction

Mydaeinae is a subfamily of Muscidae in Diptera, with more than 380 species currently known worldwide and represented by 129 species in China. They are distributed all over the world, with the majority distributed in the Oriental and Palaearctic regions. Some scholars have studied the species of the Mydaeinae, such as Hennig (1957) , Huckett (1965) , Emden (1965), Vockeroth (1972) , Pont (1977 , 1980, 1986, 1989), Shinonaga (2003) , and Carvalho (2005) , and since the 1980s, many species have been described from China and summarized by many scholars, such as Ma (1986 , 2002), Wei (1991 , 1992, 1994, 2010, 2011, 2012), Fan (1992) , Xue (1983 , 1996, 1998, 2012), Feng (2000 , 2001, 2003, 2005, 2007, 2009), and Wang (2013) . The species of Mydaeinae can be recognized by the following characters: frontal vitta without interfrontal setae; the lower margin of posterior spiracle without seta; meron bare, with thin and short hair at most, katepi sternal setae not situated with triangular; anepimeral bare, vein Sc curved as arc-shaped; post surface of hind coxa without hairs, hind tibia without *pd,* anterodorsal bristles apically long, longer than its diameter at least.

In the present paper, four new species, namely *Mydaea franzosternita***n. sp.** , *Myospila apicaliciliola***n. sp.** , *Myospila maoershanensis***n. sp.** , and *Myospila subflavipennis***n. sp.** are described. A key to genus of Mydaeinae from China and keys to species of genera from Mydaeinae are provided.

## Materials and Methods

The specimens examined for this paper were collected by sweeping from brushwood in the mountainous regions of northeast, southeast, and central China, and were collected with hand nets. Genitalic structures were detached from the body, cleared by warming in a 10% NaOH solution (approximately 100°C) for several minutes, placed in a droplet of glycerol, and observed under a compound light microscope. Terminology of external morphology and of the male terminalia follows McAlpine (1981) .

The type specimens studied in this paper are deposited in the Institute of Entomology, Shenyang Normal University, Shenyang, Liaoning Province, China.

Absolute measurements are used for the body length in millimeters (mm). Abreviations used for characters are: *ors =* orbital seta; *ori =* frontal seta; *acr =* acrostichal seta; *prst-acr =* anterior-acrostichal seta; *post-acr =* post-acrostichal seta; *dc =* dorsocentral seta; *prst-dc =* anterior dorsocentral seta; *post-dc =* posterior dorsocentral seta; *ial =* intra-alar seta; *pra =* prealar seta; *p* = posterior bristle; *v* = ventral bristles; *ad =* anterodorsal seta; *pd =* posterodorsal seta; *av =* anteroventral seta; *pv* = posteroventral seta; Sc = subcosta; Mt. = mountain.

### Nomenclature

This publication and the nomenclature it con tains have been registered in ZooBank. The LSID number is: urn:lsid:zoobank.org:pub:D2171D52-871F-4034-BA4F-32E57A141F90. It can be found online by inserting the LSID number after www.zoobank.com/ .

**Taxonomy**

### Key to genus of *Mydaeinae* from China (Males)

1. Radial node bare………………………...2 Radial node with hairs………….……….3

2. Sternite 1 with hairs………………..……. ……. *Gymnodia*Robineau-Desvoidy, 1863 Sternite 1 bare …. *Hebecnema* Schnabl, 1889

3. *Pra* present and strong…………………..4 *Pra* absent or trichoid…………………...6

4. Fore tibia without *pv* ………………...…… ……… *Mydaea*Robineau-Desvoidy, 1830 Fore tibia with *pv* ……………………..…5

5. Meron bare, sternite 1 bare………………. …………… *Sinopelta*Xue *et* Zhang, 1996 Meron with hairs, sternite 1 with hairs…... ……………. *Lasiopelta*Malloch, 1928

6. Eye kidney shape in profile.…….…..…… ….. *Graphomya*Robineau-Desvoidy, 1830 ye ovoid shape in profile *………Myospila*Robineau-Desvoidy, 1830

### Genus *Graphomya* Robineau-Desvoidy, 1830

**Type species:** *Musca maculata* Scopoli, 1845: 305.

**Generic diagnosis:** Posterior margin of eye inflexed; meron in the lower of posterior spiracle setosus, the inner of lower calypter with lobe; distal of vein M _1+2_ curving, dorsal and ventral surfaces of radial node all with setulae.

### Key to species of *Graphomya* from China (Males)

1. Species yellow in color; eye narrow, palpus yellow; legs yellow; sternite 1+2 yellow, both sides of sternite 3 in the middle without patch except median stripe…… *Gr. paucimaculata*Öuchi, 1938 Species brownish black or taupe in color; eye broad, palpus dark brown to brown; legs dark brown to brown; sternite 1+2 dark, both sides of sternite 3 in the middle with patches……………………………..2

2. *Ori* with fine hairs; the light stripe of scutum subequal with the black paramedian stripe in width; legs all black except tibiae; sternite 1+2 mostly brown…….…………. *…………………….Gr. rufitibia*Stein, 1918*Ori* with dense hairs; the white stripe of scutum shorter than the black paramedian stripe in width; legs all black; sternite 1+2 mostly dark black………………………..3

1. Body covered with white pruinosity; distal of vein M _1+2_ curving slightly; the pruinosity and stripe of the abdomen distinctly, both sides of sternite 3 in the middle with big and brown triangular patches………….. *Gr. maculata* ( Scopoli, 1763 ) Body covered with yellow pruinosity; dis tal of vein M _1+2_ curving intumescently; the pruinosity of abdomen lightly, the stripes of sternite lightly except the median stripe, both sides of sternite 3 in the middle with small and brown traversed patches………. *Gr. maculata tienmushanensis*Öuchi, 1939

### Genus *Gymnodia* Robineau-Desvoidy, 1863

**Type species:** *Gymnodia pratensis* Robineau-Desvoidy, 1863 : 635.

**Generic diagnosis:** Basisternum of prosternum always bare; basal part of vein R _4+5_ , dorsal and ventral surfaces of radial node completely bare; sternite 1 broad, the margin of sternite 1 always with setae.

### Key to species of *Gymnodia* from China (Males)

1. End of vein M _1+2_ straight………………..2 End of vein M _1+2_ curving forward………3

2. Legs all black; *prst-acr* 2; triangular stripes of tergites 3 and tergites 4 extending to two flanks…………………………. …….. *Gy. humilis* ( Zetterstedt, 1842-1860 ) Basal part of tibia yellow; *prst-acr* 4; tri angular stripes of tergites 3 and tergites 4 not extending to two flanks…………….... ……. *Gy. genurufoides* Xue *et* Wang, 1992

3. *Dc* 2+3 *Gy. polystigma* ( Meigen, 1826 ) *Dc* 2+4……………………………….….4

4. Fore tibia with *pv* 2……………………...5 - Fore tibia with *pv* 1……………...…..8

5. *Prst-acr* 4–5, the biggest space broader than the space from *prst-acr* to *post-dc* rows……………………………………..6 *Prst-acr* 2–4, the space narrower than or subequal with the space from *prst-acr* to *post-dc* rows……… …………………7

6. Eyes with dense ciliae; genal dilation very broad extending to vibrissa angle and faci al ridge; vein M _1+2_ curving forward slightly…….… *Gy. lasiopa* ( Emden, 1965 ) Eyes bare; genal dilation separates from vibrissa angle and facial ridge; vein M _1+2_ curving forward distinctly……………...… ……... *Gy. yunnanensis* Xue *et* Chen, 1992

7. Hind femur with fringe rows; tergites 5 with a pair of narrow L-shaped stripes…………… *Gy. sichuanensis* Xue *et* Feng, 1992 Hind femur without *pv* ; tergites 5 without stripe….. *Gy. latifronta* Xue *et* Wang, 1992

8. Legs brown partially………………...…… …………. *Gy. tonitrui* ( Wiedemann, 1824 ) Legs all black………….………………...9

9. Tergites 4 only with a pair of big triangu lar stripes or a pair of small stripes in the middle………………………………….10 Tergites 4 with two pairs of stripes…… ……………….. *Gy. distincta* ( Stein, 1909 )

10. Parafacial without black pruinosity, ab domen transparent, tergites 4 with a pair of big triangular stripes…………………... ……………... *Gy. ascendens* ( Stein, 1915 ) Parafacial with gray pruinosity, abdomen dark, tergites 4 with a pair of small stripes…… *Gy. nigrogrisea*Karl, 1939

### Genus *Hebecnema* Schnabl, 1887

**Type species:** *Anthomyia umbratica* Meigen, 1826 : 88.

**Generic diagnosis:** Male frons flat, antennal arista plumose; *post* - *dc* 4, *pra* absent or trichoid, notopleuron bare; vein M _1+2_ straight; hind tibia without *pd* ; abdomen without pair patches.

### Key to species of *Hebecnema* from China (Males)

1. Eyes bare………………………………...2 Eyes with hairs……………………..……4

2. Katepisternal setae 2+2; sternite 1 with hairs; cercal plate straight in pro file……………... *H. xishuicum*Feng, 2009 -Katepisternal setae 1+2; sternite 1 bare; cercal plate arc-shaped in profile………..3

3. Abdomen yellow, tegula black, calypters brown…………………………………….. ….. *H. arcuatiabdomina* Feng *et* Fan, 2001 Abdomen black, tegula brown, calypters light yellow *....H. vespertina* ( Fallén, 1823 )

4. Tibiae yellow to dark yellow……………5 Tibiae entirely black…………………….6

5. Eyes bare; calypters brown; cercal plate thin strip-shaped in profile………..……… ……………….. *H. fumosa* ( Meigen, 1826 ) Eyes with dense and long hairs; calypters white; cercal plate long cone-shaped in profile…………... *H. dasyopos*Feng, 2009

6. Calypters white………. *H. alba*Xue, 1983 Calypters light brown to brown………....7

7. Frons with a pair of *ors………………….* …. ………………… *H. manasicus*Feng, 2009 Frons without *ors* ………………………..8

8. Eyes with sparse ciliae; *ial* 0+3……… ……………. *H. umbratica* ( Meigen, 1826 ) Eyes with dense ciliae; *ial* 0+2………….9

9. Arista short plumose, sternite 1 with hairs *H. coronata* Feng *et* Wang, 2010 Arista long plumose, sternite 1 bare…...… ……………….. *H. invisifacies*Feng, 2009

### Genus *Lasiopelta*Malloch, 1928

**Type species:** *Lasiopelta orientalis* Malloch, 1928 : 309.

**Generic diagnosis:** *Pra* long and subequal with posterior notopleural seta at least, reaching to the transverse suture almost, the distance between *pra* and transverse suture shorter than 1/4 of the distance between *pra* and supra-alar seta; sternite 1 with hairs.

### Key to species of *Lasiopelta* from China (Males)

1. Basisternum of prosternum with hairs…… ……………… *L. longicornis* ( Stein, 1915 ) Basisternum of prosternum bare………...2

2. Wing dark brownish, hind tibia with 1 *ad.* ………………. *L. maculipennis*Wei, 1992 Wing hyaline, hind tibia with 2 *ad* ……...3

3. *Ori* 5, hind tibia with 4 *av* ……………...… *L. rufescenta*Wei *et* Jiang, 2010 - *Ori* 2, hind tibia with 1 *av* ………………... *…………………..L. flava*Wei *et* Cao, 2010

### Genus *Mydaea* Robineau-Desvoidy, 1830

**Type species:** *Mydaea scutellaris* Robineau-Desvoidy, 1830 : 480.

**Generic diagnosis:** Basisternum of prosternum bare; vein M _1+2_ always straight.

### Key to species to *Mydaea* from China (Males)

1. *Post-dc* 3………………………………...2 *Post-dc* 4………………………………...7

2. Femora black……………………………3 Femora yellow…………………………..4

3. Hind femur with complete and developed *pv* rows………………..……….…...…5 Hind femur without *pv…………………..* …. *…………………..Myd. shuensis*Feng, 2003

4. Parafacial about 3/5 of postpedicel in width, antennal arista as long as antennal postpedicel; *pra* absent; basicosta black; fore tibia without medial *pv* , hind femur with complete *pv* rows………………….... ……… *Myd. bideserta* Xue *et* Wang, 1992 Parafacial about 1/3 of postpedicel in width, antennal arista about 1/3 of antennal postpedicel in length; *pra* longer than posterior notopleural seta; basicosta yel low; fore tibia with 1 medial *pv* , hind femur without *pv Myd. jubiventera* Feng *et* Deng, 2001

5. Frons about 2.0 times as wide as antennal postpedicel, frontal vitta about 2.0 times as wide as fronto-orbital plate………….… …….. *Myd. laxidetrita* Xue *et* Wang, 1992 Frons subequal with antennal postpedicel in width, frontal vitta disappeared in the middle part………………………………6

6. Eyes with dense hair, frons with complete *ori* , parafacial about 2.0 times as wide as antennal postpedicel………………...……. ……… *Myd. gansuensis* (Ma *et* Wu, 1992) Eyes bare, frons with partial *ori* , parafacial about 1/2 of antennal postpedicel in width. *… **Myd. franzosternita*****Xue***et***Tian, n. sp.**

7. Scutellum yellow or basal part yellow; legs mostly yellow………………………8 Scutellum dark black; legs slightly yellow……………………………………..11

8. Hind femur with complete *pv* rows……..9 Hind femur without distinct *pv* ……………… *Myd. tinctoscutaris* Xue, 1992

9. Anterior spiracle yellow, scutellum and fore femur entirely yellow, *prst-acr* 2….... ………………... *Myd. gracilior* Xue, 1992 Anterior spiracle fuscous, neither scutellum nor fore femur yellow, *prst-acr* 1…10

10. Scutellum yellow mostly; trochanter and coxa of fore leg, basal half of fore femur and all tarsi fuscous; cerci plate broad in profile…… *Myd. setifemur*Ringdahl, 1924 Basal part of subscutellum fuscous; all legs yellow; cerci plate narrow in pro file….. *Myd. kangdinga* Xue *et* Feng, 1992

11. Hind femur yellow at least……………..12 All femora fuscous……………………..22

12. Hind femur without *pv* …………………13 Hind femur with uncompletely and trichoid *pv* row……………………………...14

13. Frons subequal with anterior ocellus in width; basicosta yellow; hind tibia with 1 *av* and 1 *ad;* abdomen with shifting patches …….…...… *Myd. discocerca*Feng, 2000 Frons about 2.0 times as wide as anterior ocellus; basicosta fuscous; hind tibia with 2(3) *av* and 2 *ad;* abdomen without shifting patch……………….……………….15

14 *. Pra* about 1/2 of posterior notopleural seta in length; wing brown, basal half of hind femur with *pv* obviously………….19 *Pra* longer than posterior notopleural seta; wing yellow, hind femur with sparse and short *pv* …………………………………20

15. Antennal arista ciliated, the longest hair subequal with antennal postpedicel in width…………………………………...16 Antennal arista short ciliated, the longest hair longer than antennal postpedicel in width…………………………………...17

16. Parafacial about 1/2 of postpedicel in width, basicosta brown, *pra* about 2/3 of posterior notopleural seta in length, hind tibia with 2 *av Myd. brevis*Wei, 1994 Parafacial subequal with postpedicel in width, basicosta dark brown, *pra* about 1.3 times as long as posterior notopleural seta, hind tibia with 3 *av………………………….. ……………Myd. fuchaoi*Xue *et* Tian, 2012

17. Frons with 7-8 pairs of *ori* …………….… ……………...… *Myd. affinis*Meade, 1891 Frons with 12-14 pairs of *ori* ………….18

18. Basisternum of prosternum yellow, *pra* subequal with posterior notopleural seta in length, metapleura with hairs; mid tibia with 2-4 *p,* hind tibia with 2-3 *av* ……….. ……………. *Myd. flavifemora*Feng, 2000 Basisternum of prosternum black, *pra* longer than posterior notopleural seta in length, metapleura bare; mid tibia with 2 *p,* hind tibia with 2 *av……………………* …. …………... *Myd. nigribasicosta*Xue, 1996

19. Frons about 2.0-2.5 times as wide as anterior ocellus; hind tibia with *2 av………….* .. …………... *Myd. brunneipennis*Wei, 1994 Frons subequal with anterior ocellus in length; hind tibia with 1 *av………………… ……………………….Myd. nigra*Wei, 1994

20. Frons with 10 pairs of *ori* ; *pra* about 1.5-2.0 times as long as posterior notopleural seta; hind tibia with *3 av* ……………. *Myd. urbana* ( Meigen, 1826 ) Frons less than 6 pairs of *ori; pra* longer than posterior notopleural seta; hind tibia with 2 *av* ……………………………….21

21. Frons with 6 pairs *of ori;* tibia dark black, mid tibia with 3-4 *p Myd. glaucina*Wei, 1994 Frons with 4-5 pairs of *ori;* tibia yellow, mid tibia with *2p Myd. minutiglaucina*Xue *et* Tian, 2012

22. Legs all black…………………………..23 Legs not all black………………………24

23. Antennal arista ciliated, the longest hair subequal with antennal postpedicel in width; *pra* about 3/4 of posterior notopleural seta; fore tibia with 1 medial *p,* hind femur with *pv;* abdomen with shifting pat-patches *Myd. sinensis* Ma, Wu *et* Cui, 1986 Antennal arista plumose, the longest hair about 1.5 times as wide as postpedicel; *pra* about 1.3 times as long as posterior notopleural seta; fore tibia without medial *p* , hind femur without *pv* ; abdomen without shifting patch………………………. *… ………………...Myd. ancilloides* Xue, 1992

24. Hind femur with 1 *pv* at least…………..25 Hind femur without *pv* …………………27

25. Only basal half of hind femur with *pv* , tib ia fuscous *………...Myd. nubila*Stein, 1916 Hind femur with complete *pv* row, tibia yellow………………………………….26

26. Frons distinct narrower than antennal postpedicel; hind tibia with 1 *av* and 2 *ad* …………….. *M. minor* Ma *et* Wu, 1986 Frons about 2.2 times as wide as antennal postpedicel; hind tibia with 2 *av* and 3 *ad* ……………... *Myd. latielecta* Xue, 1992

27. Frons wider than the distance between outer margins of posterior ocellus, the longest aristal hair about 2.0 times as wide as antennal postpedicel; tibia yellow, hind tibia with 1 *av……………………………* ….. …… *Myd. emeishanna* Feng *et* Deng, 2001 Frons narrower than the distance between outer margins of posterior ocellus, the longest aristal hair subequal with antennal postpedicel in width; tibia dark brown, hind tibia with 2(3) *av* ………………….28

28. Palpus black, mid femur with strong *a* ………………………………………….. *..Myd. jiuzhaigouensis* Feng *et* Deng, 2001 Palpus brown, mid femur without *a* …...29

29. Abdomen with white pruinosity, the cerci about 1.3 times as long as broad…………. ………………. *Myd. subelecta*Feng, 2000 Abdomen with caesious pruinosity, the length of the cerci subequal with the width……….. *Myd. scolocerca*Feng, 2000

### *Mydaea franzosternita* Xue & Tian, n. sp. ( Figure 1A–D )

**Figure 1.**
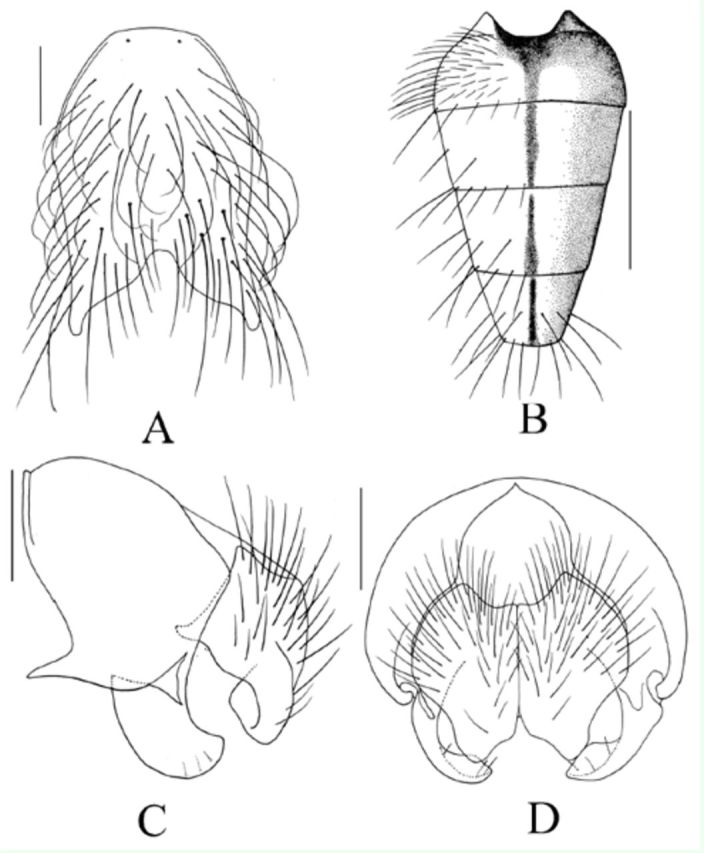
*Mydaea franzosternita* Xue *et* Tian n.sp. Male: A. Sternite 5 in ventral view, scale bar = 0.2 mm; B. Abdomen in dorsal view, scale bar = 1 mm; C. Terminalia in profile, scale bar = 0.2 mm; D. Terminalia in posterior view, scale bar = 0.2 mm. High quality figures are available online.

**Holotype Male** . Body length 7.5 mm.

***Head.*** Eyes bare; frons subequal with antennal postpedicel in width, fronto-orbital plate joint on middle part, basal half of frons with 5 pairs of *ori* ; fronto-orbital plate, parafacial and gena covered with silvery white pruinosity, parafacial about 1/2 of postpedicel in width; antennae dark brown, postpedicel about 2.0 times as long as wide, arista short plumose, the longest hair subequal with antennal postpedicel in width; epistoma not projecting in profile, vibrissal angle situated behind frontal angle in profile, genal height about 1/9 of eye height, genal and postgenal hairs all black, the upper lateral area of the occiput with hairs; proboscis short, prementum about 2.0 times as long as wide, palpi dark brown, longer than haustellum.

***Thorax.*** Black in ground color, with light grey pruinosity, presutural area of scutum with 1 black vitta, postsutural area of scutum without distinct vitta; *acr* 8, *dc* 2+3, *ial* 0+2; *pra* about 3/5 of posterior notopleural seta in length, lateral and ventral surfaces of scutellum, notopleuron, prosternum, meron and katepimeron all bare, katepisternal setae 1+2.

***Wings.*** Transparent, veins brownish, basicosta blacknish brown, costal spine shorter than crossvein rm, radial node with setulae, veins R _4+5_ and M _1+2_ all straight, vein M S-shaped; halteres yellow, calypteres yellowish.

***Legs.*** Tibiae yellow, otherwise black; fore tibia without medial *p* ; mid femur with row of 1 *pv* , becoming short apically, and with 2 apical *a* and 3 *pd* , mid tibia with 2 *p* ; hind femur with complete *av* and *pv* rows, basal half fine trichoid, and with fine *v* , hind tibia with 3 *av* , 2 *ad* , and 4(5) short *p* , without apical *pv* ; tarsi longer than tibiae, claws and pulvilli small and short.

***Abdomen.*** Black, cone-shape in dorsal view, covered with dense dark yellow pruinosity, without shifting patch; sternite 1 bare, lateral lobes of sternite 5 yellow, with strong 5–6 setae, basal part with long fringe.

***Female** .* Unknown.

**Type material.** *Holotype* : 1 male, Mt. Chang-baishan, Jilin Province, 2691 m, 15. v. 2004, collected by Chun-Tian Zhang. Holotype is deposited in IESNU. *Paratypes* : 1male, same data as holotype.

**Remarks.** This species resembles *Mydaea laxidetrita* Xue & Wang, 1992, but differs from it by having a frons subequal in width with antennal postpedicel; *pra* about 3/5 of posterior notopleural seta; lateral lobes of sternite 5 yellow, basal part with long fringe.

**Etymology.** The species name is derived from the Greek words *franza* meaning fringe and *sternita* meaning sternite, referring to the male basal part of the lobes of sternite 5 with dense and long fringes.

### Genus *Myospila* Rondani, 1856

**Type species:** *Musca meditabunda* Fabricus, 1781: 444. *Phasiophana* Brauer *et* Bergen-stamm, 1891: 390; *Trichomorellia*Stein, 1918 : 204; *Xenosia* Malloch, 1921: 421; *Xen-osina*Malloch, 1925 : 509; *Eumyiospila* Malloch, 1926: 499; *Helinella* Malloch, 1926: 498; *Pahangia*Malloch, 1928 : 311; *Eumydaea* Karl, 1935: 41; *Sinomuscina* Se-guy, 1937: 358; *Oramydaea*Snyder, 1949 : 20; *Parapictia* Pont, 1968: 179.

***Generic diagnosis* :** Vein Sc arc-shaped, dorsal and ventral surfaces of radial node with hairs (except *Myo. lenticeps* ( Thomson, 1869 ) just ventral surface with hairs), distal of Vein M _1+2_ always curving forward, the opening distance of cell 2 R _5_ about 2.0 times as long as crossvein r-m at least; hind tibia without distinct *pd* , hind coxa without hair on posterior surface.

### Key to species to *Myospila* from China

1. Basisternum of prosternum bare………...2 Basisternum of prosternum with hairs…14

2. Thorax and abdomen all yellow, apical cerci not divided in posterior view………. …………….. *Myo. xanthisma* Shinonaga *et*Huang, 2007 Thorax dark black, abdomen with yellow part at most or black, apical cerci divided in posterior view………………………...3

3. Abdomen with yellow part at most……...4 Abdomen black………………………….5

4. Hind femur blackish brown except for yel lowish brown basally ， mid femur brownish black at basal 1/2………………. ………………… *Myo. basilara*Wei, 2012 Hind femur brownish black, mid femur

brownish black at basal 2/3 *………………… ………Myo. paratrochanterata* , Wei, 2012

5. Postsutural *ial* short and small; basicosta yellow; tibia yellow; abdomen with yellow part………………………………………6 Postsutural *ial* 2, about 2.0 times as long as body hairs; basicosta dark brown; tibia black; abdomen without yellow part…….8

6. Hind tibia with 1 *av* and 2 *ad* ……………. *………………..Myo. flavipedis* Shinonaga *et*Huang, 2007 Hind tibia with 1 *av* and 1 *ad* ……………7

7. Dorsal surfaces of radial node with hairs; tarsi black, hind femur without *pv* on basal half...… *….Myo. argentata* ( Walker, 1856 ) Dorsal surface of radial node bare; tarsi yellow, hind femur with fine *pv* on basal half…………….. *Myo. boseica*Feng, 2005

8. Frons with 2 pair of *ors* ; distal of vein M _1+2_ slightly curving forward…………...9 Frons with 1 pair of *ors* ; distal of vein M _1+2_ distinct curving forward……… 11

9. Legs black to dark black mostly *…………... ……………Myo. armata*Snyder, 1940 (♀, according to Holotype) Fore femur, fore coxa and tarsi black, other parts yellow………………………….10

10. Eyes bare; basicosta balck; tergites with obvious narrow and grayish pruinosity stripes……………………………....…… ……... *Myo. breviscutellata* Xue *et* Kuang, 1992 Eyes with dense ciliae; basicosta yellow; tergites 3–5 with wide and black median stripes………….. *Myo. vernata*Feng, 2005

11. Frons with 1 pair of *ors* at least …………... *Myo. mingshanana*Feng, 2000 Frons without *ors* ………………………12

12. Eyes almost bare…………………………. …….. *Myo. meditabunda* ( Fabricius, 1781 ) Eyes with long ciliae…………………...13

13. Eye with dense ciliae, parafacial about 1/3 of antennal postpedicel in width; fore tibia with *pv* , mid tibia with 1(2) *pv* , hind tibia with 3(4) *av* ; surstyli straight in profile….. ………... *Myo. angustifrons*Malloch, 1922 Eye with sparse ciliae, parafacial subequal with antennal postpedicel in width; fore tibia without *pv* , mid tibia without *pv* , hind tibia with 2 *av* ; surstyli arc-shaped in profile………... *Myo. kangdingica*Qian, 2005

14. Lower calypter broad, the distal part inflexed……………………………….….15 Lower calypter narrow, the distal not inflexed…………………………………..17

15. Meron with hairs, abdomen with patch vittae… *Myo. bina* ( Wiedemann, 1830 ) Meron bare, abdomen without patch vittae…..…………………………………..16

16. Mid femur dark brown, tergite 3 to 4 without spot, cerci broad and short………. ………………... *Myo. binoides*Feng, 2005 Mid femur yellow, tergite 3 to 4 with spot, cerci narrow and long *………………………. ………………………Myo. longa*Wei, 2011

17. *Ial* 0+2, ventral surface of scutellum bare.. *………………………………………………* 18 *Ial* 0+1, lateral of scutellum with fine hairs on ventral surface………………………43

18. Basicosta black brown or black; coxae black to dark brown……………………19 Basicosta yellow; coxae yellowish brown…………………………………..30

19. Basicosta black………………………...20 Basicosta dark brown…………………..22

20. Trochanter brownish black…………….21 Trochanter yellow …………………… *Myo. cetera*Wei, 2012

21. Cercus narrower and surstylus straight viewed from laterally and posteriorly……. …………….. *Myo. subflavitibia*Wei, 2012 Cercus wider and surstylus curved viewed from laterally and posteriorly……………. ………… *Myo. piliungulisoides*Wei, 2012

22. Eyes with dense and long ciliae………..23 Eyes bare (few with fine hairs)………...27

23. Anterior spiracle red; hind tibia with 4(3) *ad* on apical half; tergite 3 with a pair of brown patches; tergite 5 without vitta…… …... ***Myo. apicaliciliola* Xue *et* Tian n. sp.** Anterior spiracle yellowish; hind tibia with 2 *ad* on apical half; tergite 5 with vittae………………………………………24

24. Tergite 3 with patch……………………25 Tergite 3 without patch………………...26

25. Parafacial reddish brown, frons narrow, about 1.3 times as wide as anterior ocellar-width; wings grey, hyaline……………….. ………………. *Myo. brunneusa*Wei, 2012 Parafacial black brown, frons wider, about as wide as ocellar triangle-width; wings brownish…………. *Myo.vittata*Wei, 2012

26. Mid and hind femora entirely brownish yellow *…………………………………………. ……….Myo. lasiophthalma* ( Emden, 1965 ) Mid and hind femora brownish black……. ……… *Myo.paralasiophthalma*Wei, 2012

27. Scutellum often brown; distal part of mid femur and hind femur yellow……………. ……… *Myo. trochanterata* ( Emden, 1965 ) Scutellum and femora all black………..28

28. Epistoma at the same vertical line with frontal angle in profile, palpus brown……. ………………... *Myo. subtenax*Xue, 1998 Epistoma placed behind frontal angle in profile, palpus black.……………….…..29

29. The front of metapleura with hairs, a pair vittae of scutum not reaching scutoscutellar suture, femur brown…………………... …………………. *Myo. tenax* ( Stein, 1918 ) The front of metapleura bare, a pair vittae

of scutum reaching scutoscutellar suture, femur dark black……………………… ……………. *Myo. nigrifemura*Feng, 2005

30. Vein R _1_ without seta…………………...31 At least dorsal surface of vein R _1_ with rows of setae………………… 34

31. Frons *ori* less than 14; postpronotal lobe mostly brown…………………....……..32 Frons with 14–16 pairs of *ori* ; postpronotal lobe black………………………..….33

32. *Ori* 6, notopleuron bare………………..…. *……………………Myo. laevis* ( Stein, 1900 ) *Ori* 12, notopleuron with hairs *…………………..Myo. brunnea*Feng, 2005

33. Genal and postgenal hairs all black; the inner stripe of scutum black…………………………… …...…… *Myo. maoershanensis***Xue***et***Tian n. sp.**

Genal and postgenal hairs all yellow; the inner stripe of scutum black before trans verse suture, other parts yellownish brown……….. *Myo. subbruma*Feng, 2003

34. Postpronotal lobe, scutellum and proe-pisternum all yellow; apical half of scutellum with some black setae on lateral surface; dorsal and ventral surfaces of vein R _4+5_ with fine rows of setae ………… *Myo. setipennis* ( Malloch, 1930 ) Postpronotal lobe, scutellum and proe-pisternum all balck; apical half of scutellum without setae on lateral surface; dorsal and ventral surfaces of vein R _4+5_ with fine rows of setae…………………35

35. Postpedicel brown yellow……………...36 Postpedicel black………………………38

36. Abdomen without patches……………..37 Abdomen with patches………………...… ………….. *Myo. femorata* ( Malloch, 1935 )

37. Postgena with dark setulae……………….. ………………. *Myo. flavilauda*Wei, 1991 Postgena with yellow setulae…………….. ……………… *Myo. ruficornica*Wei, 2012

38. All coxae yellow…………………………. ……………… *Myo. frigoroida*Qian, 2005 All coxae blacknish brown…………….39

39. Katepisternal setae 2+2; only basal part of vein R _1_ with 5 setae on dorsal surface…… …….. *Myo. fuscicoxoides* Xue *et* Lin, 1998 Katepisternal setae 1+2; all dorsal surface of vein R _1_ with long setae……………...40

40. Distal of cerci in posterior view, surstyli longer than cerci in profile……………..41 Distal of cerci flat, surstyli subequal with cerci in length in profile………………..42

41. Vein R _1_ bare on dorsal surface; Vein R _4+5_ with setulae on dorsal and ventral sur faces………….. *Myo. pudica* ( Stein, 1915 ) Vein R _1_ sparsely setulose on dorsal sur face; Vein R _4+5_ bare *……………………….... ……………...Myo. flavilobulusa*Wei, 2012

42. Postgena with black setulae; tarsus yellow……………. *Myo. fuscicoxa* ( Li, 1980 ) Postgena with yellow setulae; tarsus black…………….. *Myo. acrula*Wei, 2012

43. Ventral and lateral surfaces of scutellum bare……………………...………… 44 Ventral and lateral surfaces of scutellum with some fine hairs….…………….…..61

44. Scutellum brownish yellow except basal part……………….……………...……..45 Scutellum all blacknish brown…………52

45. Only fore femur black; posterior margin of sternite 5 black *………………………………. …………Myo. ateripraefemura*Feng, 2005 All femora black; posterior margin of sternite 5 brown………………………..46

46. Frons narrower than anterior ocellus in width, postpedicel always yellow; scutellum brownish yellow mostly…………….. …………….. *Myo. hainanensis*Xue, 1998 Frons subequal with anterior ocellus in width at least, postpedicel black to dark brown; scutellum yellow apically……...47

47. *Ori* not reach to front ocellus, postpronotal lobe yellow, the lateral of scutellum with hair……………………………………..48 *Ori* reach to front ocellus, postpronotal lobe black, the lateral of scutellum without hair……………………………………..50

48. Fore femur yellow, cerci shorter than surstyli in length *………………Myo. flavihumera*Feng, 2001 The 2/3 base of fore femur black, cerci subequal with surstyli in length………..49

49. Thorax brown *…...Myo. xuthosa*Wei, 2011 Thorax black……………………………... …………. *Myo. tianmushanica*Feng, 2005

50. Anterior of metanepisternum with light and short hairs; tergite 5 brownish yellow mostly………………………………….… ……. *Myo. rufomarginata* ( Malloch, 1925 ) Anterior of metanepisternum without hair; tergite 5 black mostly…………………..51

51. The anterior margin of gena with 1 row of upcurving setulae; katepisternal setae 1+2; dorsal and ventral surfaces of vein R _4+5_ with hairs; lateral of tergites 4 with a pair of light brown patches respectively…….... …………... *Myo. changzhenga*Feng, 2000 The anterior margin of gena with 3 rows of upcurving setulae; katepisternal setae 2+2; basal of vein R _4+5_ bare on ventral sur face; tergite 4 without patch……………... …………. *Myo. emeishanensis*Feng , 2005

52. Palpus dark brown or light black; femora, tibiae and trochanters brown yellow…...53 Palpus black; legs black mostly, except apical part of femora…………………...55

53. Palpus black…………………………...…. ……………. *Myo. sparsiseta* ( Stein, 1915 ) alpus dark brown……………………..54

54. Basicosta yellow……………………… ……………….. *Myo. lautoides*Feng, 2005 Basicosta black…………………………... ……………….... *Myo. sublauta*Wei, 2011

55. Basicosta black; tarsi black…………….56 Basicosta yellow to brownish yellow; tarsi yellow………...………………..… 57

56. Postpedicel about 4.0 times as long as wide; hind femur without *pv* ……………... …………... *Myo. mimelongata*Feng, 2007 Postpedicel about 2.0 times as long as wide; hind femur with 1 *pv* at least…… …………… *Myo. elongate* ( Emden, 1965 )

57. Basal half of vein R _1_ with 1 row of setae on dorsal surface.………………...… 58 Vein R _1_ bare 59

58. Scutellum blacknish brown; halteres yel low; coxae and femora dark black……….. ………….…. *Myo. guangdonga*Xue, 1998 Basal and ventral surface of scutellum yel low; halteres light red brown; coxae and femora dark yellow……….……………… ………… *Myo. frigora* Qian *et*Feng, 2005

59. Eyes with sporadic hairs, frons with 12-13 pairs of *ori……… Myo. bruma*Feng, 2003 Eyes bare, frons with 5-6 pairs of *ori* … .60

60. Pedicel yellow; scutellum with distinct median stripes; only tarsi and tibiae black…...……………………………….… ………... *Myo. flavipennis* ( Malloch, 1928 ) Pedicel black; scutellum without median stripe; legs mostly black except tibiae brownish yellow and tarsi brown………… … ***Myo. subflavipennis* Xue *et* Tian n. sp.**

61. Frons about 1/5 head in width at least, frons with 2 pairs of *ors* ………………..… ………………… *Myo. latifrons*Wei, 1991 Frons subequal with the distance between outer margins of posterior ocelli at most, frons with 1 pairs of *ors* ………………..62

62. Frontage surface of ommatidium enlarged; abdomen mostly light yellow…………..63 Frontage surface of ommatidium not enlarged; abdomen dark brown…………..64

63. Notopleuron bare, katepisternal seta 1+2... ……………………. *Myo. ponti*Xue, 1996 Notopleuron with hairs, katepisternal seta 2+2……………….. *Myo. fengi*Wei, 2011

64. Tarsi dark brown, at least fore coxa black.. …………………. *Myo. lauta* ( Stein, 1918 ) Tarsi yellow, coxae slight black……….65

*65. Ial* 1+2 *…..Myo. flavibasis* ( Malloch, 1925 ) *Ial* 1+1………………………………….66

66. Abdomen black………………………...… ……..…… *Myo. flavilauta* Xue *et* Li, 1998 The base of abdomen yellow 67

67. Frons narrow, frontal vitta disappear, antenna dark brown; posterior spiracle and femur yellow……………………………... …………. *Myo. flavibasisoides*Wei, 2011 Frons broad, frontal vitta existence, antenna black; posterior spiracle and femur dark brown………………………………...…. ……… *……..Myo. subflavibasis*Wei, 2011

### *Myospila apicaliciliola* Xue & Tian, n. sp. ( Figure 2A–D )

**Figure 2.**
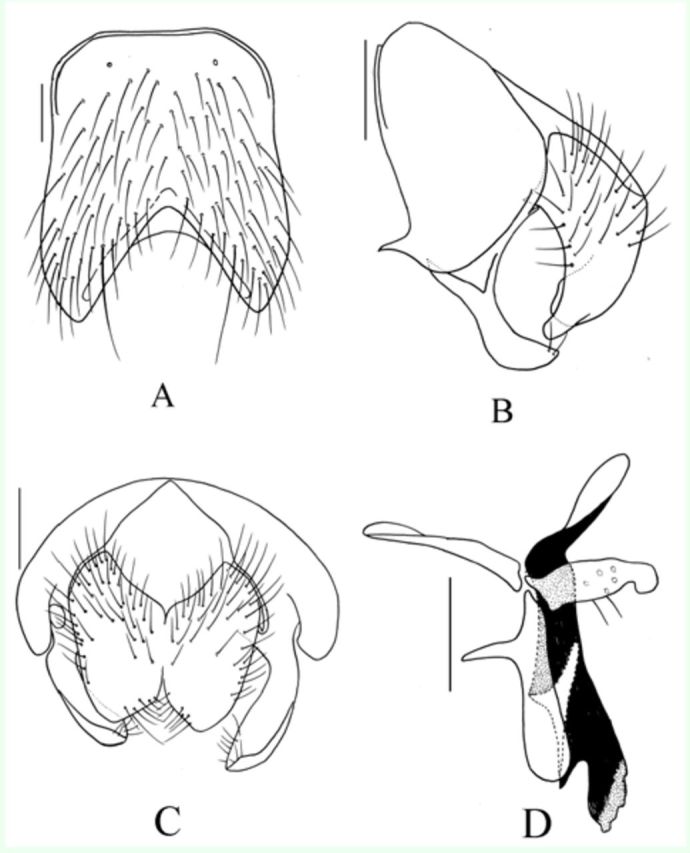
*Myospila apicaliciliola* Xue *et* Tian n.sp. Male: A. Sternite 5 in ventral view; B. Terminalia in profile; C. Terminalia in posterior view; D. *Myospila maoershanensis* Xue *et* Tian n.sp. Male: Genitalia in profile. All scale bar = 0.2 mm. High quality figures are available online.

**Holotype Male** . Body length 7.5-8.0 mm.

***Head.*** Eyes with short and dense ciliae; frons wider than the distance between outer margins of posterior ocelli, frontal vitta black, about 2.0 times as wide as fronto-orbital plate, frons with 11-13 pairs of *ori,* becoming shorter apically; both sides of anterior ocellus with a pair of retroverted *ors,* about 2.0 times as long as the upper one; fronto-orbital plate and parafacial covered with dark gray pruinosity, parafacial about 1/2 of postpedicel in width; antenna black, postpedicel about 4.0 times as long as wide, arista long plumose, the longest hair about 2.5 times as wide as aristal basal diameter; epistoma not projecting in profile, anterior margin of gena with 2 rows of upcurving subvibrissal setulae; vibrissal angle situated behind frontal angle in profile; gena slightly shining, covered with sparse brown pruinosity, genal height about 1/6 of eye height; genal and postgenal hairs entire black, the upper lateral area of the occiput with hairs; proboscis short, prementum shining, covered with sparse grey pruinosity, about 1.5 times as long as wide; palpus black, longer than the length of prementum; labella large, subequal with prementum in length.

***Thorax.*** Black in ground color, scutum covered with grayish pruinosity, postsutural area of scutum covered with grey pruinosity, between *dc* rows with a black narrow stripe; 6 rows of trichoid *prst-acr, post-acr* 1, *dc* 2+4, *ial* 0+2, *pra* short, about 1/3 posterior notopleural seta in length; notopleuron bare; scutellum brownish yellow apically, lateral and ventral surfaces bare; basisternum of prosternum with hairs, anepimeron, meron and katepimeron all bare; anterior spiracle yellow, posterior spiracle blackish brown; upper proepimeral setae 2, anterior anepisernal setae 3; katepisternal setae 2+2.

***Wings.*** Transparent; basicosta dark brown, costal spine short and small, ventral surface of vein C with hairs, vein Sc curving, arc-shaped, subcosta sclerite brown; the whole length of vein R _1_ bare, basal of vein R _4+5_ , dorsal and ventral surfaces of radial node with setulae, distal part of M _1+2_ curving forward; upper calypter brownish, lower calypter yellow and tongue-shaped, about 1.5 times as long as the upper one; halteres brownish yellow.

***Legs.*** Apical of femora yellow, other parts blackish brown; fore tibia without median *p* ; mid femur with 1 row of *a* on basal half, *pv* strong on basal half, 3(4) *pd* , short and long 1 *av* , trichoid apically, without preapical *a* , mid tibia with 3 *p* ; hind femur with 1 complete and long *av* row, without *pv* , hind tibia with 2(1) *av* and 4(3) *ad* on distal half, without apical *pv* ; hind tarsi slightly shorter than its tibia, claws and pulvilli normal, slightly shorter than tarsomere 5.

***Abdomen.*** Black, oviform, covered with grayish to brownish grey pruinosity, without glittery patch, body hairs short and dense, tergite 3 with a pair of brown elliptic patches, tergite 4 with a pair of blackish brown triangular patches, tergite 5 with a narrow faint pruinosity vitta, tergites 4 and 5 with distinct posterior marginal and discal setae, with distinct piliferous patches; sternite 1 bare, apical part of sternites 2 and 4 with strong setae.

### Female. Unknown.

**Type material.** *Holotype* : 1 male, Mt. Mao’er shan, Guangxi Zhuangzu Autonomous Region, 800 m, 16. v. 2004, collected by Ming-fu Wang. Holotype is deposited in IESNU. *Paratypes* : 2 males, same data as holotype.

**Remarks.** This species resembles *Myospila lasiophthalma* ( Emden, 1965 ), but differs from it by in tergite 5 dark brown mostly, without distinct patch; inner margin of male cerci very straight and with many small hairs apically.

**Etymology.** The species name is derived from the Latin words *cilium* meaning small ciliae, referring to the male apical part of the cerci, which has many small ciliae.

### *Myospila maoershanensis* Xue & Tian, n. sp. ( Figure 3A–D )

**Figure 3.**
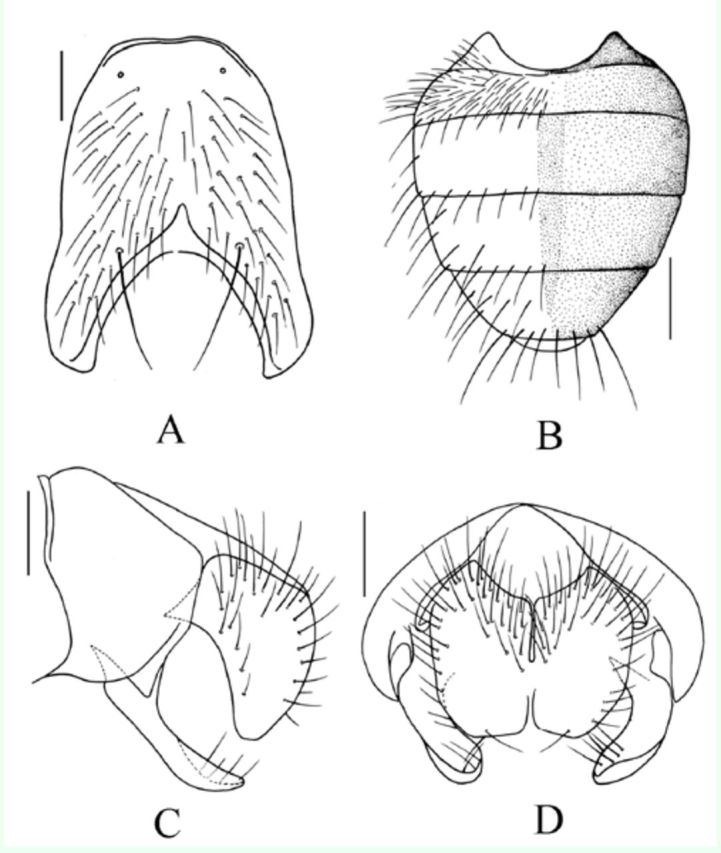
*Myospila maoershanensis* Xue *et* Tian n.sp. Male: A. Sternite 5 in ventral view, scale bar = 0.2 mm; B. Abdomen in dorsal view, scale bar = 1 mm; C. Terminalia in profile, scale bar = 0.2 mm; D. Terminalia in posterior view, scale bar = 0.2 mm. High quality figures are available online.

**Holotype Male** . Body length 7.2–7.5 mm.

***Head* .** Eyes bare; frons about 2.0 times as wide as anterior ocellus; frontal vitta black, the narrowest part wirelike, frons with 13–15 pairs of *ori* , becoming shorter toward forward, the upper one just subequal with anterior ocellus in width, front also with upper orbital seta 1, about 3.5 times as wide as anterior ocellus, fronto-orbital plate and parafacial covered with silvery white pruinosity; parafacial about 2/5 of postpedicel in width; antennae black, postpedicel about 3.5 times as long as wide, arista long plumose, the longest hair about 2.5 times as wide as aristal basal diameter; epistoma not projecting, with a row of upcurving subvibrissal setulae; vibrissal angle situated behind frontal angle in profile; gena with dark brown pruinosity, slightly shining, genal height about 1/9 of eye height; beard and postgena setae all black, dorsal area of occiput with hairs; proboscis short, prementum shining, covered with few grey pruinosity, about 1.6 times as long as wide; palpus blackish brown, longer than prementum in length.

***Thorax.*** Black in ground color, anterior of scutum covered with silvery white pruinosity, posterior of scutum covered with grey pruinosity, inner part of *dc* row with a black narrow stripe, and the outside one too wide; *acr* 0+1, *dc* 2+4, *ia* 0+2, *pra* about 2/5 of posterior notopleural seta in length; notopleuron bare; apical half of scutellum brownish yellow, lateral and ventral surfaces bare; basisternum of prosternum with hairs, anepimeron, meron and katepimeron all bare; anterior spiracle yellow, posterior spiracle dark brown; upper proepimeral setae 2, anterior anepisernal setae 1; katepisternal setae 1+2.

***Wings.*** Transparent; tegula dark brown, basicosta brownish yellow, costal spine short and small, ventral surface of vein C with hairs, vein Sc curving as arc-shaped, subcosta sclerite brownish yellow; vein R _1_ bare, base of vein R _4+5_ , dorsal and ventral surfaces of radial node all with setulae, distal of vein M _1+2_ curving forward; calypters brownish yellow, the lower calypter tongue-shaped, about 1.8 times as long as the upper calypter; halteres yellow.

***Legs.*** Femora, tibiae and trochanters yellow, coxae brownish yellow, tarsi blackish brown; fore tibia without median *p* ; mid femur with a short *av* row 2/5 of distal, 6(7) *pv* on distal half, preapical with 3 *pd* , mid tibia with 3(2) *p* , without other seta; hind femur with a complete and long *av* row, without *pv* ; hind tibia with 2 *av* (including a small *av* ), 1 median *ad* , without *pd* and apical *pv* ; all tarsi longer than tibiae, claws and pulvilli normal, slightly longer than tarsomere 5.

***Abdomen.*** Black, round-shaped, every tergite covered with a grey median pruinosity vitta, both sides of the vitta covered with sparse brownish grey pruinosity, and extended to katepisternum, without glitter patch, body hairs short and dense; posterior of tergite 3 and 4 smoke-color, but without lateral patch; posterior marginal setae on tergite 4 and 5 strong, discal setae on tergite 5 distinct; sternite 1 bare, posterior margin of sternite 2(4) with a pair of strong setae respectively.

*Female.* Unknown.

**Type material.** *Holotype* : 1 male, Mt. Mao’er shan, Guangxi Zhuangzu Autonomous Region, 800 m, 16. v. 2004, collected by Dong Zhang. Holotype is deposited in IESNU. *Paratypes* : 1 male, same data as holotype.

**Remarks.** This species resembles *Myospila subbruma*Feng 2003 , but differs from it in both beard and postgena setae yellow, inner vitta on presutural area of scutum yellow, and the one on postsutural area of scutum black; frons with 13–15 pairs of *ori* , becoming shorter toward the front, the upper one just subequal with anterior ocellus width, front it also with a upper orbital seta, about 3.5 times as wide as anterior ocellus; prementum shining, covered with few grey pruinosity, about 1.6 times as long as wide.

**Etymology.** The species is named after the locality of the holotype, referring to the species found in China: Guangxi Zhuangzu Au-Autonomous Region: Mt. Mao’er shan.

### *Myospila subflavipennis* Xue *et* Tian, n. sp. ( Figure 4A–D )

**Figure 4.**
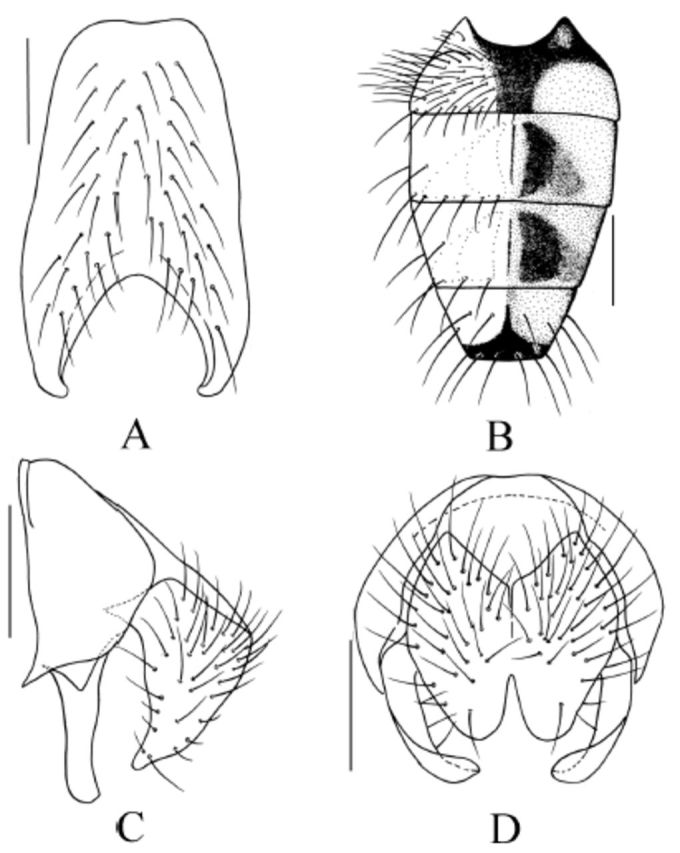
*Myospila subflavipennis* Xue *et* Tian n.sp. Male: A. Sternite 5 in ventral view, scale bar = 0.2 mm; B. Abdomen in dorsal view, scale bar = 1 mm; C. Terminalia in profile, scale bar = 0.2 mm; D. Terminalia in posterior view, scale bar = 0.2 mm. High quality figures are available online.

**Holotype Male** . Body length 5.8–6.3 mm.

***Head.*** Eyes bare; frons about subequal with the distance between outer margins of posterior ocelli; frontal vitta black, obliterated in the middle; frons with 5–6 pairs of *ori* , distributing over 3/5 lower of frons; fronto-orbital plate and parafacial covered with silvery white pruinosity; parafacial about 1/3 of postpedicel in width; antennae black, postpedicel about 3.5 times as long as wide, arista long plumose, and the longest hair about 2.5 times as wide as its basal diameter; epistoma not projecting in profile, with a row of upcurving subvibrissal setulae; vibrissal angle situated behind frontal angle in profile; gena covered with grey pruinosity, slightly shining, genal height about 1/6 of eye height; beard and postgena setae all black, dorsal area of occiput with hairs; proboscis short, prementum shining and covered with few grey pruinosity, about 2.0 times as long as wide; palpus blackish brown, longer than prementum in length, labella large, subequal with prementum in length.

***Thorax.*** Black in ground color, anterior of scutum covered with silvery white pruinosity, posterior of scutum covered with grey to brownish grey pruinosity, inner part of *dc* row with a black narrow stripe, and the outside one too wide; *acr* 0+1, *dc* 2+4, *ia* 0+1, *pra* trichoid, about 1/3 of posterior notopleural seta in length; notopleuron bare; scutellum the same color as thorax, lateral and ventral surfaces of scutellum bare; basisternum of prosternum with hairs, anepimeron, meron, and katepimeron all bare; spiracles dark brown; just with upper proepimeral setae long and large 1, anterior anepisernal seta 1; katepisternal setae 1+2.

***Wings.*** Transparent; tegula and basicosta black, costal spine short and small, ventral surface of vein C with hairs, vein Sc curved as arc-shaped, and subcosta sclerite brownish yellow; vein R _4+5_ base, dorsal and ventral surfaces of radial node bare, distal of vein M _1+2_ curved forward; calypters brown, the lower calypter tongue-shaped, about 1.8 times as long as the upper calypter; halteres yellow.

***Legs.*** Blackish brown except tiabiae brownish yellow, tarsi brown; fore tibia without median *p* ; mid femur with a short *av* row 2/5 of distal, long 3(4) *pv* on distal half, preapical with 3 *pd* , mid tibia with 3 *p* , without other seta; hind femur with a complete of short *av* row, and just 4(5) large apically, without distinct *pv* ; hind tibia with 2 *av* , median with 1 *ad* , without *pd* and apical *pv* ; all tarsi longer than tibiae, claws and pulvilli small, about 1/2 of tarsomere 5 in length.

***Abdomen.*** Black, egg-shaped, covered with dense grey pruinosity, without glittery patch, tergite 3 and 4 with a wide brown trapezia-shaped patch respectively, in the middle of the patch with a brownish grey pruinosity vitta, divided the trapezia-shaped patch into two indistinct parts; posterior marginal setae and discal setae on tergite 4 and 5 thick and large, posterior margin of tergite 5 shining and brown; black median part narrow; sternite 1 bare, posterior margin of sternite 2(4) with a pair of strong setae respectively, sternite 5 lanky.

*Female.* Unknown.

**Type material.** *Holotype* : 1 male, Mt. Jianfeng, Gougu Rain forest, Hainan Province, 500–1000 m, 19.v.2004, collected by Ming-fu Wang. Holotype is deposited in IESNU. *Paratypes* : 6 males, same data as holotype.

**Remarks.** This species resembles *Myospila flavipennis* ( Malloch, 1928 ), but differs from it by the pedicel being black; scutellum without a broad median vitta; legs entirely black except tibiae brownish yellow and tarsi brown.

**Etymology.** The species name is from the Latin words *sub* meaning resemble, referring to the male resemblance to the species *Myospila flavipennis* ( Malloch, 1928 ).

### Genus *Sinopelta* Xue *et* Zhang, 1996

Type species: *Sinopelta latifrons*Xue *et* Zhang, 1996 : 202.

*Generic diagnosis:* Male frons broad; scutum without *acr,* katepi sternal setae 2+2, meron bare; mid tibia with *pv,* hind tibia with 2 *av;* lateral lobe of sternite 5 with strong bunchy setae.

### Key to species of *Sinopelta* from China (Males)

1. With *ors,* epistoma projecting; prementum about 6.0 times as long as height; halteres black; fore tibia with 1 medial *pv,* mid tibia with 2 *ad…………………………………… ……………S. latifrons*Xue *et* Zhang, 1996 Without *ors,* epistoma not projecting, prementum about 2.5 times as long as height; halteres brown yellow; fore tibia without medial *pv,* mid tibia without *ad* … *.* …….. *S. maculiventra*Xue *et* Zhang, 1996
